# Genome-Wide Association Study for Cytokines and Immunoglobulin G in Swine

**DOI:** 10.1371/journal.pone.0074846

**Published:** 2013-10-02

**Authors:** Xin Lu, JianFeng Liu, WeiXuan Fu, JiaPeng Zhou, YanRu Luo, XiangDong Ding, Yang Liu, Qin Zhang

**Affiliations:** 1 Key Laboratory Animal Genetics, Breeding and Reproduction, Ministry of Agriculture, College of Animal Science and Technology, China Agricultural University, Beijing, China; 2 Collaborative Innovation Center for Diagnosis and Treatment of Infectious Diseases, State Key Laboratory for Infectious Disease Prevention and Control, National Institute for Communicable Disease Control and Prevention, Chinese Center for Disease Control and Prevention, Beijing, China; 3 Department of Animal and Food Sciences, University of Delaware, Newark, Delaware, United States of America; University of California, Davis, United States of America

## Abstract

Increased disease resistance through improved immune capacity would be beneficial for the welfare and productivity of farm animals. To identify genomic regions responsible for immune capacity traits in swine, a genome-wide association study was conducted. In total, 675 pigs were included. At 21 days of age, all piglets were vaccinated with modified live classical swine fever vaccine. Blood samples were sampled when the piglets were 20 and 35 days of age, respectively. Four traits, including Interferon-gamma (IFN-γ) and Interleukin 10 (IL-10) levels, the ratio of IFN-γ to IL-10 and Immunoglobulin G (IgG) blocking percentage to CSFV in serum were measured. All the samples were genotyped for 62,163 single nucleotide polymorphisms (SNP) using the Illumina porcineSNP60k BeadChip. After quality control, 46,079 SNPs were selected for association tests based on a single-locus regression model. To tackle the issue of multiple testing, 10,000 permutations were performed to determine the chromosome-wise and genome-wise significance level. In total, 32 SNPs with chromosome-wise significance level (including 4 SNPs with genome-wise significance level) were identified. These SNPs account for 3.23% to 13.81% of the total phenotypic variance individually. For the four traits, the numbers of significant SNPs range from 5 to 15, which jointly account for 37.52%, 82.94%, 26.74% and 24.16% of the total phenotypic variance of IFN-γ, IL-10, IFN-γ/IL-10, and IgG, respectively. Several significant SNPs are located within the QTL regions reported in previous studies. Furthermore, several significant SNPs fall into the regions which harbour a number of known immunity-related genes. Results herein lay a preliminary foundation for further identifying the causal mutations affecting swine immune capacity in follow-up studies.

## Introduction

Increasing robustness by improving resistance/tolerance to pathogens is an important selection objective in animal breeding. In the past 30 years, selection for growth, carcass leanness, meat quality and prolificacy has been highly effective in pigs [1]. Indeed, animals highly selected for production traits may be more susceptible to pathogens or less able to maintain performance after infection. In this context, including health traits in existing breeding schemes using indirect strategies is an emerging trend in pig breeding [2]. The immune system plays essential roles in disease resistance of animals. Enhancing immune capacity of animals can be goal of breeding for disease resistance.

Cytokines are important mediators in the regulation and activation of the adaptive immune response in various infections, inflammation, and even cancer development [3]. The levels of a set of cytokines, such as Interferons and Interleukin, in serum vary with health and disease status. Among them, IFN-γ and IL-10 are known to play a role in defense against virus [4], [5]. IFN-γ is an activator of the cytotoxic T cell pathway [6]. The importance of IFN-γ in the immune system is due to its ability to inhibit viral replication directly [7]. The suppression of IFN-γ response will cause the enhancement of secondary infection especially virus infection, such as porcine reproductive and respiratory syndrome virus (PRRSV), porcine circovirus type 2, and swine influenza virus [8], [9], [10]. IL-10 has pleiotropic effects on immunoregulation and inflammation. IL-10 inhibits a broad spectrum of cellular responses, including suppressing the function of APCs and T cells by inhibiting co-stimulation, MHC class II expression, and chemokine secretion [11]. Although the in vivo role of IL-10 is generally immunosuppressive, it plays an important stimulatory role in the function of B-lymphocytes and the production of antibodies by B1 lymphocytes during the development of an immune response against antigens from pathogens [12]. IL-10 down-regulates the production of pro-inflammatory cytokines and generally protects the animal from systemic inflammation [13]. The stimulatory effect of IL-10 on B cells can enhance antibody production and induce Ig-class switching and plasma cell differentiation [14]. Increased amounts of IL-10 inhibit the action of monocytes, macrophages, and NK cells during the immune response to viral infection and inhibit the synthesis of proinflammatory cytokines [12].

There are a positive feedback of IFN-γ and IL-10 on their own production and a negative control of each other's production [15]. The ratio of IFN-γ/IL-10 production reflects the capacity to activate or inhibit monocytic and T lymphocytic functions, and a higher ratio has also been shown to be associated with depressive disorders [16]. In human, it has been shown that atopic diseases, such as asthma and allergies, are associated with a pronounced skewing of the Th1/Th2-balance in the Th2-direction [17], and the susceptibility to autoimmune and infectious diseases is associated with the capacity the polarized Th1/Th2-type immune responses [18]. Schulte *et al*. (1997) found that different inbred strains of rats and mice were extremely different in their capacity of producing Th1 and Th2-type cytokines, which caused them to be different in susceptibility to different kinds of diseases, such as diabetes, experimental autoimmune encephalomyelitis (an animal model for multiple sclerosis), rheumatoid arthritis and infectious diseases (Mycobacteria) [19], [20], [21], [22]. Thus, a special focus has been placed on the skewing of the Th1/Th2-balance of the immune system. In swine, Diaza *et al*. (2003) reported that a Th1-inclined cytokine profile leading to an exacerbated local inflammation at the early installation stage of the cysticercus may interfere with their successful establishment in the serum antibodies against total cysticercus antigens [23].

Immunoglobulin G (IgG) is important in immune responses. IgG antibodies are involved in predominantly the secondary immune response. IgG is the most common immunoglobulins circulating in the blood. The presence of specific IgG corresponds to maturation of the antibody response [24]. IgG can bind to many kinds of pathogens (such as viruses, bacteria, fungi and so on), and protects the body against them by agglutination and immobilization, complement activation (classical pathway), opsonization for phagocytosis, and neutralization of their toxins [25].

In order to include immunocompetence in selection for improved health, a major challenge is to find the key genes controlling immune traits in animals with inter-individual variability in response to various pathogens. Up to now, a large amount of QTLs for immune traits have been detected and mapped to different pig chromosomes (Animal QTLdb, http://www.animalgenome.org/cgi-bin/QTLdb/index). However, the resolution of these QTLs are generally low with confidence interval 20∼30 cM and the identification of the relevant genes and quantitative trait mutations (QTMs) remains great challenge although a few prominent successful cases have been reported [26].

Recently, the first high-density 60 K porcine SNP array has been developed [27], which offers the prerequisite for genome-wide association study (GWAS), a powerful approach for high-resolution mapping of loci controlling complex traits. Using this array, a few GWA studies have been performed in pigs for androstenone levels [28], body composition and structural soundness [29], Escherichia coli F4ab/F4ac susceptibility [30], hematological traits [31], and T lymphocyte subpopulations [32]. Up to now, GWAS have been becoming a most commonly-used strategy for gene identification for complex traits in animals as well as humans.

In this study, we performed a GWAS for IFN-γ and IL-10 levels, the ratio of IFN-γ to IL-10 and IgG blocking percentage to CSFV in swine based on the swine 60 K SNP array. A suite of significant SNPs associated with these immune traits at either the genome-wise or chromosome-wise were identified. These promising SNPs may be considered as a preliminary foundation for further replication studies and eventually unraveling the causal mutations in swine.

## Materials and Methods

### Animal resource

 The animal resource used in this study consists of 562 piglets from three different breeds (Landrace, *n* = 68; Yorkshire, *n* = 415 and a Chinese indigenous breed named Songliao Black, *n* = 79). The structure of experimental population was given in Table 1. All individuals were raised from 2007 to 2009 under standard indoor conditions. At 21 days of age, all piglets were vaccinated with 4 doses live Classical Swine Fever Virus (CSF) Vaccine (Rabbit origin, tissue virus ≥0.01 mg/dose) (Qilu Animal Health Products Co., Ltd., Shandong, China) through intramuscular injection. The first blood samples were collected from each piglet one day before the vaccination (day 20), and two weeks after the vaccination, the second blood samples were collected (day 35). All blood samples were directly injected into VACUETTE® Serum Clot Activator tubes. In addition, ear tissues of all individuals were also collected. The whole procedure for collection of the samples (blood and ear tissue) was carried out in strict accordance with the protocol approved by the Animal Welfare Committee of China Agricultural University (Permit number: DK996).

**Table 1:** Constitution of the study population.

Breed	Sires	Dams	Piglets	Total
Landrace	4	13	68	85
Yorkshire	16	63	415	494
Songliao Black	3	14	79	96
Total	23	90	562	675

### Measurement of phenotypes

IFN-γ and IL-10 levels in each serum sample were measured using a commercial ELISA kit (Biosource, Carlsbad, California) according to the manufacturer's instructions. All samples were arranged randomly in each plate and a standard curve was fitted for each plate and used to calculate IFN-γ and IL-10 concentrations in each serum sample.

IgG blocking percentage in the serum was measured using the commercial CSF virus antibody test kit (IDEXX laboratories, Liebefeld-Bern, Switzerland) according to the manufacturer's instructions.

### Genotyping

 DNA was extracted from ear tissue samples of all pigs, including piglets and parental individuals. DNA was quantified and genotyped using the Illumina PorcineSNP60 BeadChip containing 62,163 SNPs, which is a multi-sample genotyping panel powered by Illumina's InfiniumH II Assay. Features of the Illumina PorcineSNP60 BeadChip have been detailed previously [27]. All samples were genotyped using BeadStudio (Illumina) and a custom cluster file developed from all samples.

### Genotype quality control

To assess the technical reliability of the genotyping panel, a randomly selected DNA sample was genotyped twice and over 99% identity of called genotypes (two mismatches) was obtained. This demonstrates the technically robust feature of the 60 K SNP BeadChip panel employed herein. All the samples included are with a minimum of 95% call rate.

Quality control procedures were as follows. First, only samples with a minimum of 90% call rate were included. Second, out of the initial full-set of 62,163 SNPs, we discarded: (1) SNPs with a call rate <90% (n = 3,812); (2) those deviating from Hardy–Weinberg equilibrium (HWE) in controls (P<10–6, n = 6,849); and (3) those having a minor allele frequency (MAF) <03 in the total sample (n = 8,274). Therefore, 46,079 SNPs were available for the subsequent analyses, and the distribution is presented in Table 2.

**Table 2:** Distribution of SNPs after quality control on each chromosome.

Chr.	No. SNPs	Average distance (kb)^a^
1	4858	60.82
2	2535	55.22
3	2161	57.05
4	2835	48.08
5	1854	54.23
6	2172	56.64
7	2672	50.92
8	1872	63.28
9	2483	53.36
10	1194	54.97
11	1539	51.86
12	1180	48.65
13	2800	51.84
14	3245	45.76
15	2053	65.43
16	1336	57.84
17	1248	51.36
18	990	54.77
23	911	137.98
0^b^	6141	NA
TOTAL	46079	

a Derived from the most recent porcine genome sequence assembly (Sscrofa9.2) (http://www.ensembl.org/Sus_scrofa/Info/Index).

b These SNPs are not assigned to any chromosomes.

### Statistical analyses

#### Mixed model based single locus regression analyses (MMRA)

Similar to the study of GWAS before [33], we performed association test for each SNP via regression analysis based on the following linear mixed model:
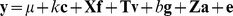
Where 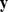 is the vector of phenotypic observations of all piglets on day 35; 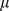 is the overall mean; 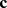 is the vector of corresponding observations of all piglet on day 20, 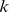 is the regression coefficient of the phenotypes on day 35 on those on day 20; 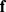 is the vector of fixed effects, including effects of breed and batch of sampling, 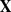 is the incidence matrix of 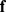; 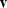 is the vector of random litter effects, 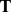 is the incidence matrix of 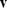; 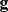 is the vector of the SNP genotype indicators which take values 0, 1 or 2 corresponding to the three genotypes 11, 12 and 22 (assuming 2 is the allele with a minor frequency), 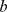 is the regression coefficient of 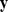 on 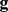. 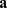 is the vector of residual polygenic effects with 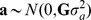 (where 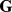 is the genomic relationship matrix constructed based on SNP markers according to VanRaden [34] and 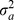 is the additive variance), 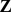 is the incidence matrix of 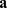; 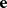 is the vector of residual errors with 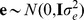 (where 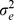 is the residual error variance).

The variance components involved in the model were estimated by using the REML method and the software DMU [35]. For each SNP, the estimate of *b* and the corresponding sampling variances 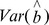 was obtained via mixed model equations (MME). A Wald Chi-squared statistic 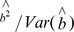 with *df*  = 1 was constructed to examine whether the SNP is associated with the trait.

 The effect of a SNP on a trait was measured as proportion of the phenotypic variance of the trait explained by the SNP. The phenotypic variance was estimated based on the model described above with the SNP genotype vector excluded. The variance explained by a SNP was calculated as 
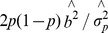
, where 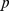 is the allele frequency of the SNP in the study population and 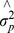 is the estimate of the phenotypic variance.

We employed Fortran 95 to code the computing program for the method and it is available upon request.

### Statistical inference

For the analyses above, the permutation method was adopted to generate the empirical distribution of the test statistic and to adjust for multiple testing from the number of SNPs tested as well. In our method, the phenotypes of all individuals were randomly resampled 10,000 times without replacement along with all their related fixed and random effects except SNP genotypes. The critical region was formed by the greatest values of the test statistics in 10000 permutation tests. The genome-wise significance was determined from the critical region which was formed by the greatest values of the test statistic among all tested SNPs in the whole genome from each of the 10,000 permutations. The chromosome-wise significance was determined in the same way except that the critical region was build for each chromosome respectively, i.e., the highest test statistic values among the SNPs on the chromosome were picked up to form the critical region. We declared a genome-wise (chromosome-wise) significant SNP if its raw test statistic value was larger than the 95^th^ percentile value of the genome-wise (chromosome-wise) empirical distribution.

### Linkage disequilibrium analyses

 Linkage disequilibrium (LD) block analyses were performed for the chromosomal regions with multiple significant SNPs clustered to further pinpoint potential candidate genes. The LD levels were detected using Haploview (Version 4.2) [36], and the LD blocks were defined by the criteria of Gabriel et al. [37].

## Results

### Alterations of the IgG and cytokine levels in peripheral blood after challenge

The descriptive statistics of IgG and cytokine concentration in peripheral blood on day 20 (the day before vaccination) and day 35 (the day two weeks after vaccination) are shown in Table 3.

**Table 3:** Descriptive statistics of the traits in the study population.

Trait	Test Day	Mean	Standard Deviation
IFN-γ (pg/ml)	20	24.5	54.75
	35	26.45	60.02
IL-10 (pg/ml)	20	94.24	193.69
	35	70.34	130.21
IFN-γ/IL-10	20	1.36	2.2
	35	1.32	2.05
IgG (%)	20	42	22
	35	29	19

Compared with the measurements on day 20, the IL-10 and the IgG concentration in blood on day 35 decreased obviously while the IFN-γ concentration in blood on day 35 increased. However, the ratio of IFN-γ to IL-10 on day 35 changed only slightly after challenge.

### Significant SNPs

**Figure 1:**
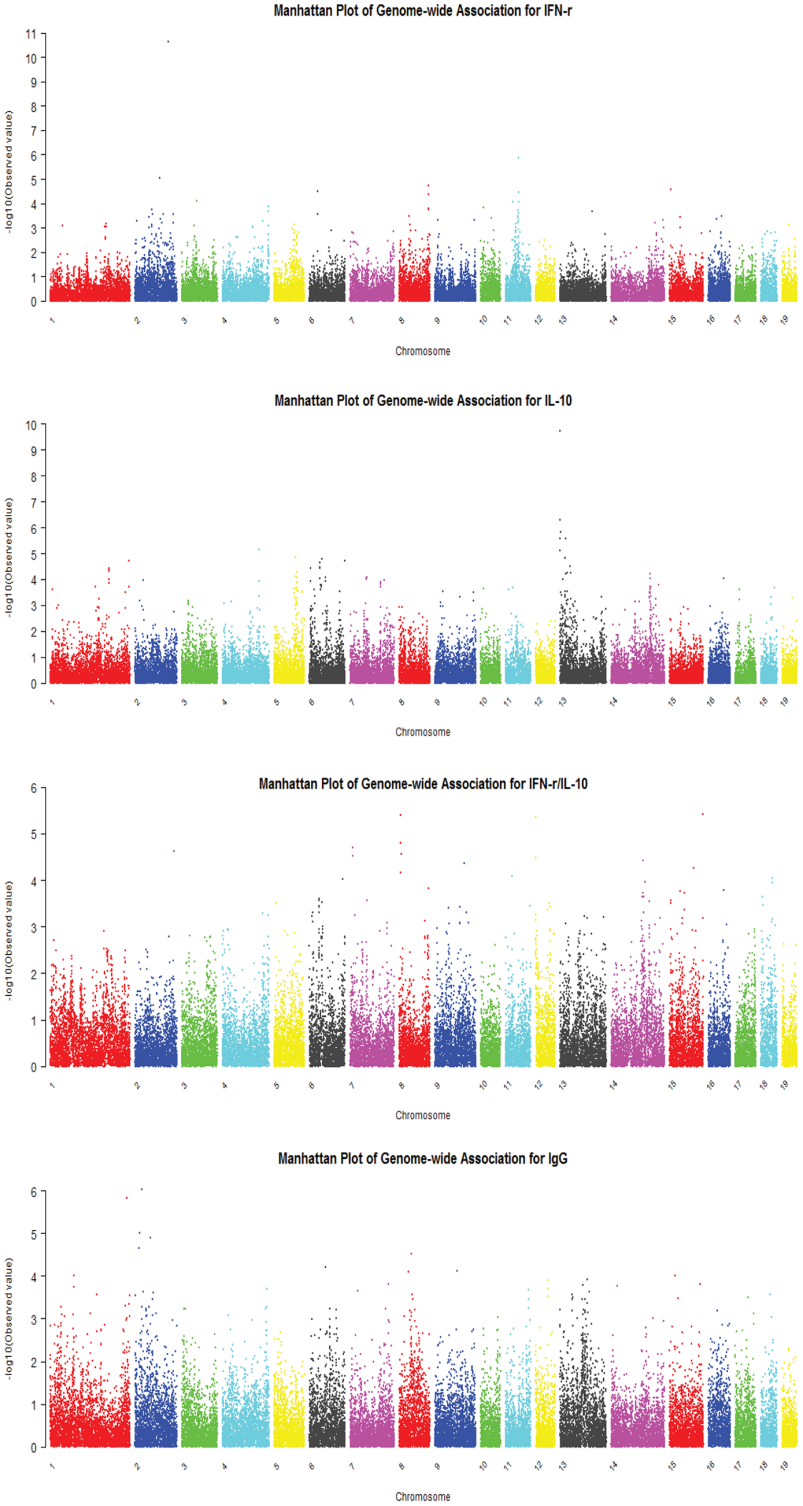
Manhattan plots of the P values of all tested SNPs (in terms of –log_10_P) for the four traits studied. Different chromosomes are represented by different colors. Chromosome 19 stands for the X chromosome of swine.

The profiles of the *P* values (in terms of –log_10_P) of all tested SNPs for the four traits are shown in Fig 1. Both genome-wise significant and chromosome-wise significant SNPs detected by MMRA for the four traits are presented in Table 4. In total, 32 significant (P<0.05) SNPs at chromosome-wise level, including 4 SNPs at genome-wise level were detected by permutation test.

**Table 4:** Significant SNPs for the IgG and cytokine concentration in peripheral blood.

Trait	No. SNPs	SNP name	SSC	Position (bp)^a^	Test statistic value^b^	Effect^c^ (%)	Nearest gene	
							Name	Distance (bp)
IFN-γ	6	DRGA0003229	2	90034957	19.6814^*^	3.23	NR2F1	234654
		MARC0043455	2	114103546	44.743116^**^	13.81	SNCAIP	171906
		ALGA0050019	8	114783076	18.27096^*^	5.13	WDFY3	107553
		H3GA0031875	11	43825030	17.16906^*^	4.96	DACH1	433007
		MARC0074652	11	44210118	17.194204^*^	4.04	MZT1	7241
		MARC0105416	11	45376499	23.3076^*^	6.35	KLF12	115100
IL-10	15	ASGA0021867	4	112459225	20.12712^*^	5.44	CTTNBP2NL	5112
		MARC0004505	5	72149862	18.85106^*^	4.75	ANO6	58736
		ALGA0035367	6	40753020	18.57244^*^	5.52	GP6	47172
		ASGA0028260	6	41123535	18.57244^*^	5.52	FCAR	48795
		ASGA0028261	6	41214511	18.57244^*^	5.52	BRSK1	21263
		MARC0045838	6	41495658	18.57244^*^	5.52	SNORD34	16890
		ASGA0083917	6	41751998	18.57244^*^	5.52	NLRP4	49397
		MARC0005355	6	42270490	18.57244^*^	5.52	ZNF582	46854
		MARC0035429	13	346105	25.14739^*^	6.92	CPNE4	204370
		ALGA0067348	13	502458	19.93306^*^	4.09	CPNE4	48017
		ASGA0055542	13	654439	40.62093^**^	7.81	CPNE4	within
		DRGA0011841	13	666272	22.0959^*^	4.54	CPNE4	within
		ASGA0055625	13	2175901	23.18236^*^	4.80	HACL1	30943
		H3GA0035652	13	13557477	18.78095^*^	6.09	RBMS3	16719
		ASGA0056401	13	13863246	22.05002^*^	5.41	RBMS3	within
IFN-γ/ IL-10	5	ALGA0046197	8	5183140	18.59629^*^	5.22	RAB28	466201
		ASGA0037649	8	5722464	21.27879^*^	6.58	RAB28	1005525
		ASGA0052467	12	954501	17.20405^*^	3.74	NPTX1	50584
		M1GA0015746	12	980805	21.03734^*^	4.72	NPTX1	24280
		ALGA0088446	15	133867757	21.37182^*^	6.48	RIF1	5366
IgG	6	MARC0056499	1	284245367	23.17908^**^	4.09	PPP2R4	140444
		ASGA0009314	2	13446316	17.93413^*^	3.75	C11orf49	190612
		ALGA0123968	2	16094605	19.57043^*^	3.74	TSPAN18	136413
		ALGA0012590	2	21923489	24.07067^**^	5.50	ALX4	5383745
		SIRI0001360	2	44793993	19.02854^*^	3.26	GALNTL4	134251
		H3GA0024856	8	39537664	17.40828^*^	3.83	PDGFC	103431

a: Derived from the recent porcine genome sequence assembly (Sscrofa9.2) (http://www.ncbi.nlm.nih.gov/).

b: * indicates chromosome-wise significant; ** indicates genome-wise significant.

c: Phenotypic variance explained by the SNP.

 For the IFN-γ concentration, 6 significant SNPs, including one at genome-wise significance level, were identified, and three of them are harbored in a narrow region (43.83 to 45.38 Mb) on SSC11. For the IL-10 concentration, 15 significant SNPs (including one SNP at genome-wise significance level) were identified. Six of them are harbored in a narrow region (40.75 to 42.27 Mb) on SSC6 and 4 of them are harbored in a narrow region (0.35 to 0.67 Mb) on SSC 13. LD analysis showed that SNPs in each of the two regions were in one LD block, respectively. For the ratio of IFN-γ to IL-10, 5 and 2 significant SNPs were identified on SSC8 and SSC12, respectively. For the IgG concentration, 6 significant SNPs (including two at genome-wise significance level) were found, and four of them are on SSC2.

## Discussion

GWAS has been considered as a promising tool for gene identification for complex traits. So far GWAS for domestic animal are largely focused on economically important growth and production traits, such as milk production in dairy cattle, backfat in swine, etc. In this study, we carried out a GWAS to explore potential causal genes for the IgG and cytokine in swine. To our knowledge, this is the first study aiming at unrevealing the genetic mechanism of those immune traits in swine based on a high density SNP chip panel. Heritability estimates of the cytokine (IL10 and IFN-γ) levels were moderate to high after PMAIONO and CONA stimulations (h^2^ = 0.41 to 1.0) [2], [38]. Heritability estimates for IgG, calculated by paternal half-sib correlation, ranged from 0.31 to 0.27 [39], indicating that selection for increased serum IgG concentrations would be possible. Therefore, as a category of immune-related traits with moderate heritability, these immune traits can be potentially implemented to selection for disease resistance and susceptibility in swine breeding. Genetic correlation estimates among IFN-γ, IL10 and IgG were generally weak (r_g_<0.3) [2], that illustrated these traits provide more or less independent potential clues for selecting for improved immunocompetence. Based on heritability and correlation estimations in Flori and collaborators' study, these immune traits might be incorporated into selection schemes, provided they are associated with improved global health and do not exhibit strong antagonisms with other economically important traits.

 In this study, we treated breed as a fixed effect to avoid potential confounding between effects of SNP and breed. And the main purpose of our study is to detect common SNPs influencing the cytokines and IgG level in serum, so we did not put the interaction effect in our association model in GWAS.

32 significant SNPs with chromosome-wise level were detected to be associated with the four traits investigated, 4 of them reached genome-wise level. These SNPs account for 3.23% to 13.81% of the total phenotypic variance individually. For the four traits, the numbers of significant SNPs range from 5 to 15, which jointly account for 37.52%, 82.94%, 26.74% and 24.16% of the total phenotypic variance of IFN-γ, IL-10, IFN-γ/IL-10, and IgG, respectively. However, some of the significant SNPs for a trait are very close to each other and may represent one SNP. Therefore, the total effect of the significant SNPs for a trait should be much less than the sum of the individual SNP effects. For example, of the 15 significant SNPs for IL-10, four are located in the region of 0.35 Mb to 0.67 Mb on SSC13 and six in the region of 40.7 Mb to 42.2 Mb on SSC6. LD analysis revealed that they are in one LD block, respectively. In particular, the 6 SNPs on SSC6 are in complete LD, suggesting their effects on IL-10 may be due to a single gene in this region. Indeed, there is only one immune-related gene, *IL-11* (interleukin 11), in this region, which can be considered as a promising candidate gene, although it is not the nearest gene to any of the 6 SNPs.

Of the significant SNPs, 17 fall into QTL regions of immune-related traits previously reported, furthermore, some of the QTL are responsible for the same traits considered in the present study. Specifically, on SSC11, the three significant SNPs with effect on the IFN-γ concentration are located within the reported QTL region for IFN-γ [40]. The significant SNP for the IL-10 concentration on SSC5 is located within the reported QTL for the IFN-γ concentration [40]. The two significant SNPs for the IgG concentration on SSC2 fall in the regions which have been reported to harbor QTL for IgG in our previous study (submitted to journal). IFN-γ regulates neutrophil activation and enhances neutrophil surface receptor expression [41] and is produced by Th1 CD4 and CD8 cytotoxic T lymphocyte effector T cells once antigen-specific immunity develops [7]. The significant SNP for the IFN-γ concentration on SSC8 is located within the reported QTL for monocyte [42]. IL-10 is a pleiotropic cytokine produced by both lymphocytes and mononuclear phagocytes [43]{Rentzos, 2009 #263}{Rentzos, 2009 #263}. The significant SNPs for the IL-10 concentration on SSC4 are located within the reported QTL for monocyte and T lymphocyte [42], [44]. All six significant SNPs for IL-10 on SSC6 are located within the QTL for T lymphocyte [44]. The two significant SNPs for IFN-γ/IL-10 on SSC12 are located within the QTL for lymphocyte [42]. The significant SNP for the IgG concentration on SSC1 is located within the QTL for the eosinophils and leukocyte [42]. These findings suggest that these regions can be considered as candidate regions for further exploring the potential functional genes in the follow-up studies.

In addition to the *IL-11* gene harbored in the LD block on SSC6, there are several other significant SNPs which fell into the regions harboring known immune-related genes (not necessarily the nearest to the SNPs). For IFN-γ, the significant SNPs (H3GA0031875, MARC0074652, and MARC0105416) were found in the region which harbors the *PIBF1* (progesterone immunomodulatory binding factor 1) gene and the *KLF5* (Kruppel-like factor 5 (intestinal)) gene. For IgG, the significant SNP (ASGA0009314) was found in the region which harbors the *MADD* (MAP-kinase activating death domain) gene, the *SPI1* (spleen focus forming virus (SFFV) proviral integration oncogene spi1) gene and the *NR1H3* (nuclear receptor subfamily 1, group H, member 3) gene. On SSC1, the SNP with genome-wise significance level (MARC0056499) for IgG falls in the region which harbors the *LRRC8A* (leucine rich repeat containing 8 family, member A) gene. The *LRRC8* family genes encode components of the pre-B cell receptor or proteins that are activated by crosslinking of the pre-B cell receptor. Defects in these genes result in a block in B-cell differentiation at the pro-B to pre-B cell transition [45], [46]. IgG molecules are synthesized and secreted by plasma B cells. Therefore, *LRRC8A* can be considered as a candidate gene for IgG in the further studies.

## Conclusions

In summary, our study revealed 32 SNPs associated with four traits (including Interferon-gamma (IFN-γ) and Interleukin 10 (IL-10) levels, the ratio of IFN-γ to IL-10 and Immunoglobulin G (IgG) blocking percentage to CSFV in serum) at chromosome-wise significance level of which 4 reached genome-wise significance level. These SNPs account for 3.23% to 13.81% of the total phenotypic variance individually. 17 of them are located within the immune-related QTL regions reported in previous studies. Furthermore, 11 of them fall into the regions harboring known immunity-related genes. Findings herein lay a preliminary foundation for further identifying the causal mutations affecting swine immune capacity in follow-up studies.
